# Establishment of a Duplex Quantitative PCR Assay for the Detection and Differentiation of African Swine Fever Virus Genotype I, Genotype II, and Genotype I/II Recombinants

**DOI:** 10.3390/v18060677

**Published:** 2026-06-17

**Authors:** Naoki Yoshida, Shiho Oka, Anh Duc Truong, Mizuki Watanabe, Mitsutaka Ikezawa, Hien Thi Thu Nguyen, Le Thi Hai Vo, Tuong Dinh Nguyen, Tomoya Kitamura, Tatsuya Nishi, Takehiro Kokuho, Hoang Vu Dang, Ha Thi Thanh Tran, Kentaro Masujin

**Affiliations:** 1 Kodaira Research Station, National Institute of Animal Health, National Agriculture and Food Research Organization, 6-20-1 Josui-honcho, Kodaira 187-0022, Tokyo, Japan; yoshida.naoki389@naro.go.jp (N.Y.); watanabe.mizuki595@naro.go.jp (M.W.); ikezawa.mitsutaka109@naro.go.jp (M.I.); kitamura.tomoya063@naro.go.jp (T.K.); nishi.tatsuya047@naro.go.jp (T.N.); kokuho.takehiro756@naro.go.jp (T.K.); 2 Saitama Prefectural Chuo Livestock Hygiene Service Center, 107-1 Bessho-cho, Kita-ku, Saitama 331-0821, Saitama, Japan; oka.shiho.am@pref.saitama.lg.jp; 3 Department of Veterinary Immunology and Epidemiology, Vietnam Institute of Animal and Veterinary Science, 86, Truong Chinh, Kim Lien, Hanoi 100000, Vietnam; truonganhduc84@gmail.com (A.D.T.); nguyenthithuhien@naue.edu.vn (H.T.T.N.); dangvuhoang@nivr.gov.vn (H.V.D.); 4 Faculty of Agriculture, Forestry and Fisheries, Nghe An University, 51 Ly Tu Trong, Ha Huy Tap, Vinh 460000, Vietnam; levth@nau.edu.vn (L.T.H.V.); tuongnd@nau.edu.vn (T.D.N.)

**Keywords:** African swine fever virus (ASFV), duplex quantitative PCR, genotype I/II recombinant ASFV, *CP2475L*

## Abstract

African swine fever (ASF) is a highly fatal, febrile infectious disease of domestic pigs and wild boars caused by the African swine fever virus (ASFV). Recently, highly virulent recombinant ASFVs with chimeric genomes derived from p72 genotype I and II viruses have emerged in China, Vietnam, and Russia. These genotype I/II recombinants can evade immunity induced by genotype II–based vaccines, thereby complicating disease control efforts. To address this challenge, a novel duplex quantitative PCR (qPCR) assay was developed to simultaneously detect and differentiate genotypes I, II, and I/II recombinants in a single reaction. The assay exhibited high sensitivity and specificity, with a reliable detection limit of 10 copies/reaction for genotype I and II ASFV DNA. Validation using clinical samples collected in northern Vietnam in 2025 confirmed a robust performance in accurately distinguishing circulating genotype II viruses from recombinant genotype I/II viruses, including the detection of potential co-infection. Whole-genome sequencing of selected positive samples further corroborated these findings. Overall, this qPCR assay provides a precise and efficient tool for identifying currently circulating ASFV genotypes, thereby facilitating improved disease surveillance and supporting a comprehensive understanding of the evolving epidemiological landscape of ASF in regions with increasing viral genetic diversity.

## 1. Introduction

African swine fever (ASF) is an acute and highly lethal hemorrhagic disease of domestic pigs and wild boars and is classified as a notifiable animal disease by the World Organization for Animal Health [1]. The causative agent, African swine fever virus (ASFV; *Asfivirus haemorrhagiae*), is an enveloped double-stranded DNA virus belonging to the family *Asfarviridae*. Its genome is approximately 170–190 kbp in length and encodes more than 150 proteins [2]. Based on the nucleotide sequence of the *B646L*, which encodes the major capsid protein, p72, ASFV isolates have been classified into at least 23 distinct genotypes [3,4].

Since the introduction of a highly virulent genotype II ASFV into the Caucasus region in 2007, ASFV has progressively expanded across Europe and Asia [5,6,7,8,9]. In China, a genotype II isolate was first detected in 2018 and subsequently spread to most Asian countries by the end of 2025 [10,11,12]. More recently, highly virulent recombinant ASFV isolates with chimeric genomes derived from genotype I and genotype II viruses (ASFV genotype I/II) have emerged in China and subsequently expanded beyond national borders into Vietnam and Russia [13,14,15]. Importantly, these recombinant viruses may evade immunity induced by genotype II viruses [13,14], including vaccine-derived immunity. Consequently, they may continue to circulate and spread, even in regions where ASFV genotype II is already endemic. Therefore, accurate characterization of circulating ASFV strains is essential for epidemiological assessment and the development of effective control strategies.

Several quantitative PCR (qPCR) assays have been developed to differentiate genotype I, genotype II, and genotype I/II recombinant ASFVs [16,17]. These assays have demonstrated high sensitivity, and the binding regions of their genotype-specific primers and probes are highly conserved among the target genotypes. However, naturally occurring deletions of these target genes have been identified in non-target genotypes [2,18], suggesting that these genes may not be essential for ASFV and that similar deletions could potentially emerge in the future.

Therefore, we focused on another target gene, *CP2475L*, which encodes the structural precursor protein pp220, an essential protein for core shell assembly, and is located within the genotype-dependently conserved central region of the ASFV genome [19]. In this study, genotype I- and genotype II-specific primer–probe sets targeting *CP2475L* were selected for a novel duplex qPCR assay that enables precise discrimination of genotypes I, II, and I/II recombinant ASFVs in a single reaction, and its performance was evaluated using field-collected samples. This diagnostic approach may facilitate detailed monitoring of the epidemiological dynamics of circulating wild-type and recombinant ASFVs and may serve as a valuable tool for strengthening ASF surveillance and control strategies under conditions of increasing viral genetic complexity.

## 2. Materials and Methods

### 2.1. Ethical Statement

All experiments involving ASFV in this study were conducted in the institute’s biosafety level 3 facility accredited by the Ministry of Agriculture, Forestry and Fisheries of Japan. All recombinant DNA experiments were approved by the National Agriculture and Food Research Organization under approval number S-23-012 on 4 July 2023. Sample collection from pigs in the field was authorized by the Ministry of Agriculture and Rural Development (MARD) of Vietnam under TCVN 8402:2010 and TCVN 8400-41:2019, with approval granted on 31 July 2024.

### 2.2. Primers and Probes

**Figure 1:**
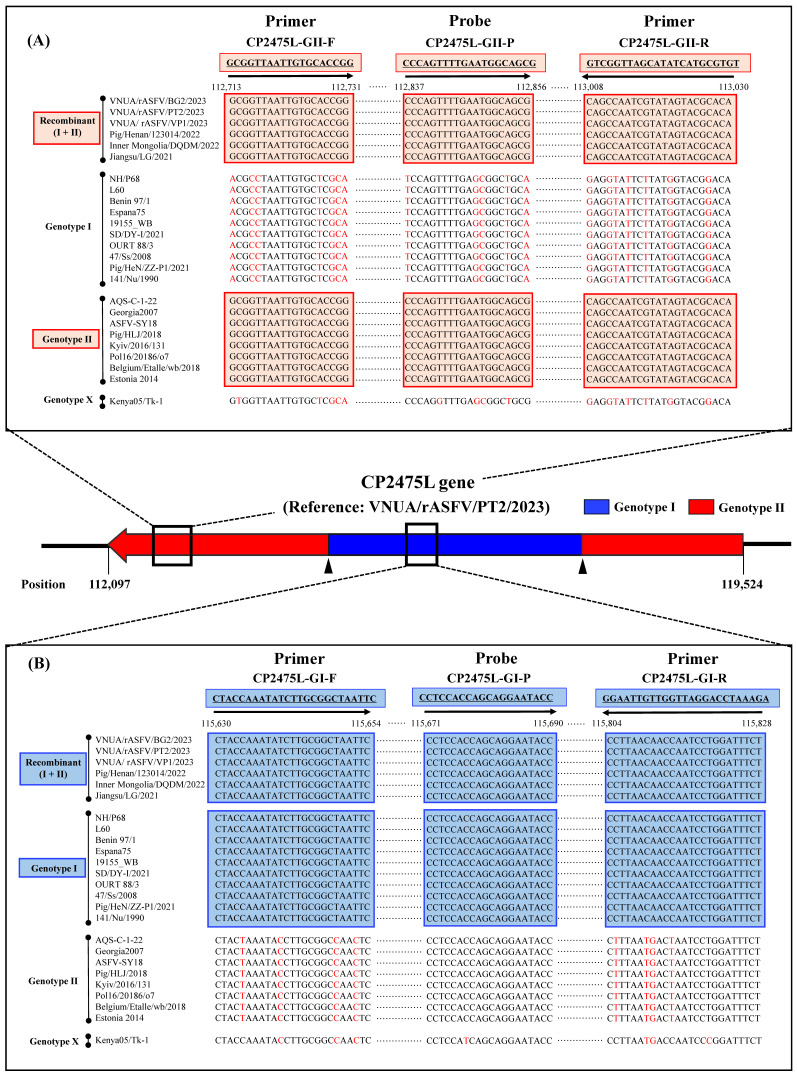
Schematic representation of the *CP2475L* in the recombinant ASFV strain VNUA/rASFV/PT2/2023 (GenBank accession no. PP810980) and positions of the duplex qPCR primer–probe sets. The *CP2475L* region in this strain comprises two genotype II–derived flanking regions (112,097–114,683 bp and 117,648–119,524 bp) and a central genotype I–derived region (114,684–117,647 bp), shown as boxes. Panel (**A**) shows the genotype II/recombinant–specific primer–probe set, along with alignments of the corresponding genome sequences from registered genotype I (10 strains), II (8 strains), genotype I/II recombinant viruses (11 strains) and one genotype X strain. Panel (**B**) similarly shows the genotype I/recombinant–specific primer–probe set. The primer and probe binding regions are shaded to highlight their positions in the genotype I, genotype II, and recombinant ASFV sequences. Red letters indicate mismatched nucleotides compared with the corresponding primer or probe sequences. Arrowheads indicate putative recombination breakpoints.

In previously reported ASFV genotype I/II recombinants [15], precise identification of recombination breakpoints has been challenging; however, two recombination sites have been identified within the *CP2475L*, resulting in a chimeric sequence derived from genotypes I and II (Figure 1). To amplify the respective genotype-specific target regions, specific primers and probes (Figure 1; Table 1) were designed based on the intact *CP2475L* sequences of reference ASFV genotypes I and II, as well as ASFV genotype I/II (Table 2). The designed primer and probe sequences were well conserved among the reference strains listed in Supplementary Table S1, comprising 84 genotype I strains, 204 genotype II strains, and 11 genotype I/II recombinant strains. All labeled and unlabeled oligonucleotides were synthesized by Eurofins Genomics (Tokyo, Japan) and stored in nuclease-free water at −20 °C until use.

### 2.3. Viruses

ASFV field isolates Armenia07 (genotype II), E75 (genotype I; GenBank accession no. NC_044958), and Kenya05/Tk-1 (genotype X; GenBank accession no. NC_044945) were kindly provided by Dr. Sánchez-Vizcaíno (Universidad Complutense de Madrid, Madrid, Spain). AQS-C-1-22 (genotype II; GenBank accession no. LC659087) was isolated from a contaminated pork product confiscated at an international airport in Japan [20]. These ASFV isolates were propagated in immortalized porcine kidney macrophage (IPKM) cell cultures and used for subsequent experiments [21,22]. IPKM cells were routinely maintained in Dulbecco’s modified Eagle’s medium (Nakalai Tesque, Kyoto, Japan) supplemented with 10% fetal bovine serum, 10 μg/mL bovine insulin (Merck, Darmstadt, Germany), 25 μM monothioglycerol (Wako, Osaka, Japan), and antibiotics. Cells were cultured in plastic plates and flasks designed for suspension culture (Sumitomo Bakelite, Tokyo, Japan).

### 2.4. Plasmids as Standards for qPCR Assays

Standard plasmids were constructed using the pGEM-T Easy vector as the backbone. The qPCR target regions of the *CP2475L*, derived from E75 (nucleotides 117,908–118,225) and AQS-C-1-22 (nucleotides 114,607–114,805), were amplified through PCR and individually cloned into the pGEM-T Easy vector. The resulting genotype-specific standard plasmids were designated pGEM-TV-GI (genotype I) and pGEM-TV-GII (genotype II), respectively, and were stored at −20 °C until use.

### 2.5. Optimization of the Duplex qPCR Assay

To optimize the duplex qPCR assay, a range of reaction conditions was systematically evaluated. Primer concentrations were tested within the range of 0.05–0.20 µM, while final probe concentrations were evaluated within 0.08–0.40 µM in a final reaction volume of 25 µL. Additionally, annealing temperatures between 56 °C and 60 °C were examined. Optimization was performed to minimize quantification cycle values while maximizing fluorescence signal intensity.

The optimized 25 μL reaction mixture consisted of 12.5 μL of 2× enzyme mix (Probe qPCR Mix MultiPlus; TaKaRa Bio, Shiga, Japan), 0.5 μL of genotype I primers (10 μM), 0.5 μL of genotype II primers (10 μM), 0.2 μL of the genotype I–specific probe (10 μM), 0.5 μL of the genotype II-specific probe (10 μM), 2 μL of template DNA, and 7.8 μL of distilled water. The duplex qPCR assay was performed using a Thermal Cycler Dice Real-Time System IV (TaKaRa Bio) under the following cycling conditions: initial denaturation at 95 °C for 20 s, followed by 45 cycles of denaturation at 95 °C for 1 s, and annealing/extension at 60 °C for 30 s. Fluorescence signals were recorded at the end of each extension step.

### 2.6. Analytical Sensitivity, Specificity and Reproducibility

To construct standard curves for the duplex qPCR assay, the two standard plasmids, pGEM-TV-GI and pGEM-TV-GII, were mixed at equal copy numbers and subjected to 10-fold serial dilutions. The mixed plasmid standards were diluted from 10^0^ to 10^6^ copies per reaction and used as templates to construct the standard curves. Each dilution was tested in triplicate in an experiment.

The analytical specificity of the duplex qPCR assay was evaluated using genomic DNA extracted from culture supernatants of E75, Armenia07, and Kenya05/Tk-1, as well as from a mixed viral sample containing E75 and Armenia07. DNA extraction was performed using the High Pure Viral Nucleic Acid Kit (Roche Diagnostics, Basel, Switzerland) in accordance with the manufacturer’s instructions. Each sample was also tested in triplicate.

Intra- and inter-assay reproducibility was evaluated by using 10^2^, 10^4^, and 10^6^ copies of the mixed plasmid standards. For intra-assay evaluation, each dilution was tested in triplicate within a single run using the established duplex qPCR assay. For inter-assay evaluation, each dilution was tested in three independent experiments performed on different days, with triplicate reactions at each dilution in each experiment. The coefficients of variation (CVs) of the cycle threshold (Ct) values were calculated based on the intra-assay and inter-assay results.

### 2.7. Ct Value Comparison of Genotype-Specific Targets Using a Synthetic Recombinant Control

To evaluate the Ct value differences between the genotype I- and genotype II-specific targets when both target regions are present on the same template molecule, a synthetic *CP2475L* fragment was prepared as a surrogate recombinant control. The fragment corresponded to nucleotides 112,580–115,896 of VNUA/rASFV/PT2/2023 (GenBank accession no. PP810980) and contained both genotype specific target regions. The synthetic fragment was serially diluted in 10-fold steps from 10^1^ to 10^6^ copies per reaction. Each dilution was tested in triplicate, and the experiment was independently repeated three times. The difference in Ct values between the two targets was calculated as ΔCt = Ct value (genotype II; Ct _genotype II_) − Ct value (genotype I; Ct _genotype I_).

### 2.8. Clinical Sample Testing

**Figure 2:**
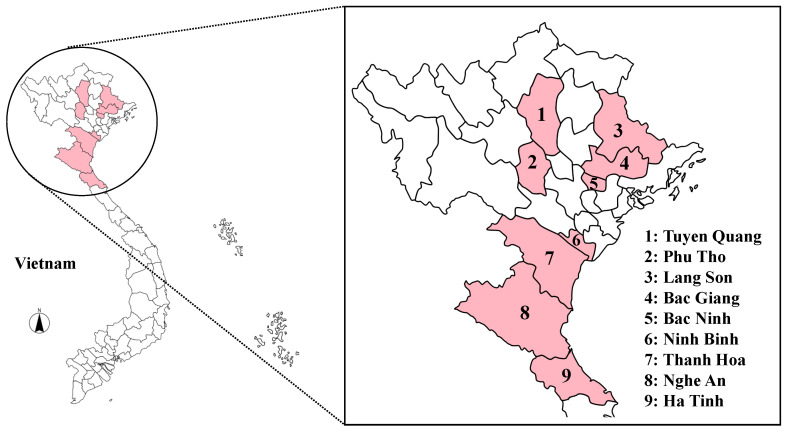
Geographic distribution of clinical sample collection sites in northern Vietnam. Pink-shaded areas indicate the provinces where samples were collected.

A total of 20 field samples were collected from ASF-affected pig farms in the northern provinces of Vietnam between July and October 2025 (Figure 2). All samples were submitted to the Vietnam Institute of Animal and Veterinary Science (formerly the National Institute of Veterinary Research) in Vietnam for diagnostic and research purposes. Viral nucleic acids were extracted from 200 μL of each sample using the High Pure Viral Nucleic Acid Kit (Roche Diagnostics) in accordance with the manufacturer’s instructions. Subsequently, 2 μL of the purified DNA was used for ASFV screening by qPCR to determine the positive or negative status of the clinical samples [23]. ASFV-positive samples were then subjected to genotyping analysis using the duplex qPCR assay developed in this study. All qPCR assays were performed using a QuantStudio™ 5 Real-Time PCR System (Thermo Fisher Scientific, Waltham, MA, USA).

### 2.9. Whole-Genome Sequencing Analysis

To validate the performance of the duplex qPCR assay, ASFV-positive clinical samples identified as ASFV recombinant–positive by the assay were subjected to whole-genome sequencing. Viral genomic DNA was amplified using a tiled PCR strategy that covers the entire ASFV genome, dividing it into multiple overlapping fragments [24]. The primers used for tiled PCR amplification are listed in Table S2. Amplicons from each sample were pooled and purified using the FastGene™ Gel/PCR Extraction Kit (NIPPON Genetics, Tokyo, Japan) before library preparation.

Sequencing libraries were constructed from the pooled amplicons using the Ion Xpress™ Plus Fragment Library Kit (Thermo Fisher Scientific) in accordance with the manufacturer’s instructions. Individual libraries were barcoded using the Ion Xpress™ Barcode Adapters 1–16 Kit (Thermo Fisher Scientific). Template preparation and chip loading were performed using the Ion 540™ Kit–Chef on the Ion Chef™ System (Thermo Fisher Scientific), and sequencing was conducted on an Ion GeneStudio™ S5 System (Thermo Fisher Scientific) using an Ion 540™ Chip, according to the manufacturer’s protocols. Sequence reads were mapped to the reference genome of the ASFV recombinant strain VNUA/rASFV/PT2/2023 (GenBank accession no. PP810980) using the Ion S5 Plus Torrent Server.

### 2.10. Phylogenetic Analysis

The phylogenetic tree was constructed from whole-genome sequence data for ASFV. Whole-genome sequences of representative ASFV strains were retrieved from the GenBank database. Multiple sequence alignment was performed using MAFFT version 7.0 on the Galaxy platform with default parameters [25,26].

Phylogenetic relationships were inferred using the maximum-likelihood method implemented in MEGA version 12.1 [27]. The optimal nucleotide substitution model was selected using the Bayesian information criterion [28] using the model selection function in MEGA, and the general time-reversible model with a proportion of invariant sites (GTR + I) was identified as the best-fitting model [29,30,31]. Alignment positions with gaps or missing data were partially excluded using a site coverage cutoff of 95%. The robustness of the inferred phylogenetic tree was assessed using bootstrap analysis with 1000 replicates.

## 3. Results

### 3.1. Evaluation of Analytical Sensitivity, Specificity and Reproducibility

**Figure 3:**
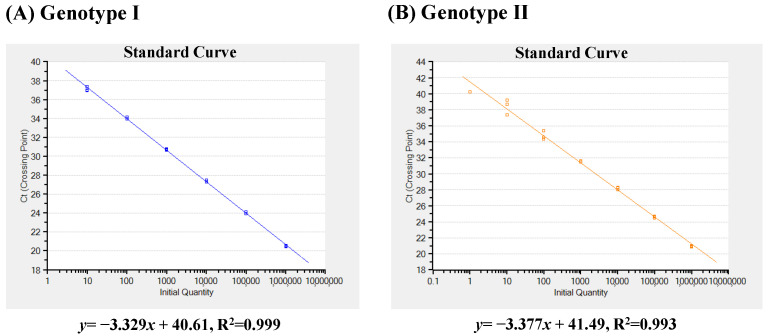
Standard amplification curves of the duplex qPCR assay generated using 10-fold serial dilutions of a positive control plasmid mixture containing equal copy numbers (10^0^ to 10^6^ copies per reaction) of pGEM-TV-GI and pGEM-TV-GII. Panel (**A**) shows the standard curve obtained using the genotype I–specific primer–probe set, and Panel (**B**) shows the standard curve using the genotype II-specific primer–probe set.

The analytical sensitivity of the duplex qPCR assay was evaluated using 10-fold serial dilutions of mixed plasmid standards (pGEM-TV-GI and pGEM-TV-GII), spanning a concentration range of 10^0^ to 10^6^ copies per reaction. Standard amplification curves for genotypes I and II are shown in Figure 3. For genotype I, the assay produced a slope of −3.329, a correlation coefficient (R^2^) of 0.999, and an amplification efficiency of 99.7%. For genotype II, the corresponding values were −3.377 for the slope, 0.993 for R^2^, and 97.8% for amplification efficiency. Reliable amplification was consistently observed for both genotypes, down to 10 copies per reaction, with all three replicates yielding positive signals. At this concentration, Ct values were 37.1 ± 0.5 for genotype I- and 38.4 ± 1.9 for genotype II (mean ± 2SD, *n* = 3; Table S3). Based on these results, the detection thresholds were set at Ct ≤ 37 for genotype I- and at Ct ≤ 39 for genotype II.

**Figure 4:**
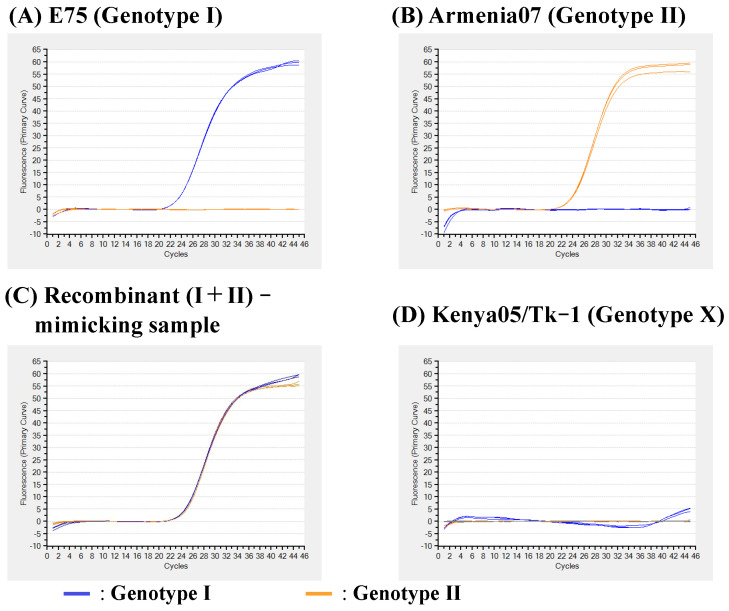
Specificity of the duplex qPCR assay. (**A**) E75 (genotype I), (**B**) Armenia07 (genotype II), (**C**) recombinant-mimicking sample (a mixture of E75 and Armenia07), and (**D**) Kenya05/Tk-1 (genotype X). E75 and Armenia07 each showed single-target amplification, whereas the mixed sample exhibited dual-target amplification. No amplification was observed for Kenya05/Tk-1.

To evaluate the analytical specificity of the duplex qPCR assay, nucleic acids extracted from E75 and Armenia07 were used as templates. Additionally, a recombinant-mimicking sample was prepared by mixing equal copy numbers of nucleic acids from E75 and Armenia07 and was subsequently subjected to analysis. Nucleic acids extracted from Kenya05/Tk-1 were also included in the analysis. Amplification of E75 resulted in the specific detection of the genotype I target, with no amplification observed for the genotype II target (Figure 4A). Conversely, amplification of Armenia07 yielded a genotype II-specific reaction, with no corresponding amplification of the genotype I target (Figure 4B). The recombinant-mimicking sample exhibited amplification of both genotype I- and II–specific targets (Figure 4C). No amplification was detected for either genotype I– or II–specific targets in the Kenya05/Tk-1 sample (Figure 4D).

To assess the reproducibility of the duplex qPCR assay, mixed plasmid standards were evaluated at different concentrations (10^2^, 10^4^, and 10^6^ copies/reaction). The results are shown in Table 3. The intra-assay CVs ranged from 0.37% to 0.96% for genotype I- and 0.20% to 0.99% for genotype II. The inter-assay CVs ranged from 0.71% to 1.21% for genotype I- and 0.28% to 1.15% for genotype II. Overall, the CVs ranged from 0.20% to 1.21%, demonstrating good reproducibility of the duplex qPCR assay.

### 3.2. Amplification Efficiency of Genotype I- and Genotype II-Specific Targets in a Synthetic Recombinant Control

The synthetic *CP2475L* fragment was used to determine whether the genotype I- and genotype II-specific targets showed comparable Ct values when both target regions present on the same template molecule. Across the 10-fold serial dilutions from 10^1^ to 10^6^ copies per reaction, both genotype-specific targets were consistently amplified. The Ct values of the two targets were similar across the tested concentrations, with a ΔCt [Ct _genotype II_ − Ct _genotype I_] value of 0.74 ± 0.66 (mean ± 3SD; Table S4). These results indicate that a single recombinant-like template produces comparable Ct values for the two genotype-specific targets. Based on the mean ± 3SD range obtained using this synthetic recombinant control, samples with ΔCt values outside the range of 0.08 (mean − 3SD) to 1.40 (mean + 3SD) were judged in this assay to represent co-infection with two distinct ASFV strains.

### 3.3. Genotyping Results of Clinical Samples

Clinical field samples collected from ASF-affected pig farms in northern Vietnam were screened to identify ASFV-positive samples using a previously reported qPCR assay [23]. Of the 20 samples analyzed, 17 were identified as ASFV-positive (Table 4). These 17 positive samples were analyzed using the duplex qPCR assay. Among them, 15 samples showed simultaneous amplification of genotype I- and II–specific targets, indicating the presence of recombinant ASFV. Among these 15 samples, two (VN/TH/62/2025 and VN/TH/03/2025) showed ΔCt values of −5.2 (Ct _genotype II_: 19.0 − Ct _genotype I_: 24.2) and −6.9 (Ct _genotype II_: 19.6 − Ct _genotype I_: 26.5), respectively, which were outside the range observed for the synthetic recombinant control. Therefore, these samples were interpreted as representing co-infection with two distinct ASFV strains in this assay. The remaining two samples were interpreted as genotype II positive based on amplification within the defined detection threshold (Table 4).

### 3.4. Whole-Genome Sequencing

Of the 15 samples identified as recombinant ASFV-positive, 10 samples—VN/TQ/97/2025 (GenBank accession no. LC921664), VN/LS/100/2025 (GenBank accession no. LC921665), VN/NA/117/2025 (GenBank accession no. LC921666), VN/NA/118/2025 (GenBank accession no. LC921667), VN/PT/31/2025 (GenBank accession no. LC921670), VN/LS/08/2025 (GenBank accession no. LC921668), VN/PT/20/2025 (GenBank accession no. LC921669), VN/BN/124/2025 (GenBank accession no. LC921671), VN/PT/21/2025 (GenBank accession no. LC921672), and VN/LS/102/2025 (GenBank accession no. LC921673)—produced overlapping PCR amplicons spanning the full viral genome using the tiled PCR approach. These 10 samples were therefore subjected to whole-genome analysis. The remaining five samples were excluded from the analysis because some genomic regions could not be successfully amplified with tiled PCR.

**Figure 5:**
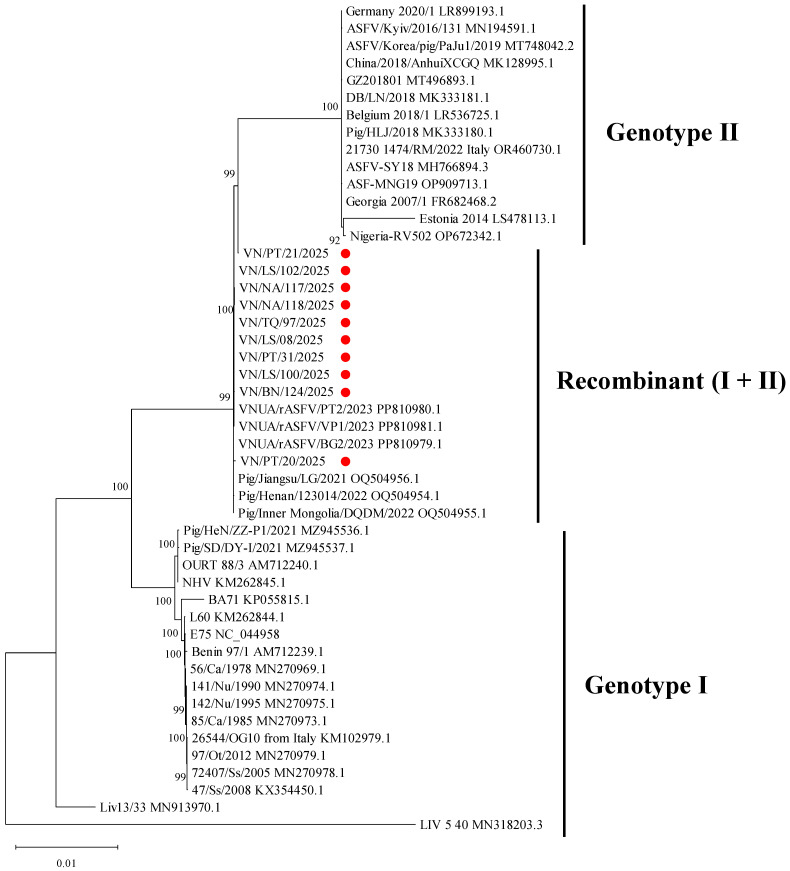
Phylogenetic analysis of ASFV whole-genome sequences. A maximum-likelihood phylogenetic tree was constructed from whole-genome sequences of ASFV using the GTR + I model with 1000 bootstrap replicates. The 10 recombinant ASFVs analyzed in this study (red dots) clustered within the genotype I/II recombinant group. Bootstrap values are shown at major nodes. The scale bar indicates the number of substitutions per site.

Genome-wide phylogenetic analysis of pooled amplicons derived from 10 recombinant ASFVs revealed that they clustered with recently reported genotype I/II recombinant strains (Figure 5). All analyzed genomic regions exhibited a high nucleotide sequence identity (99.93–99.99%) to the reference recombinant strain VNUA/rASFV/PT2/2023 (GenBank accession no. PP810980), which was isolated in northern Vietnam in 2023 (Table S5), despite the presence of several additional single-nucleotide variants (Table S6). Moreover, the viruses VN/PT/20/2025, VN/PT/21/2025, and VN/LS/08/2025 shared similar deletions within the *MGF505-7R* and *MGF505-8R* gene regions located in the LVR. Furthermore, VN/LS/08/2025 harbored additional deletions across multiple genes, including *KP86R*, *KP93L*, *MGF360-1L*, and *MGF360-2L* (Table S6).

## 4. Discussion

To date, ASF vaccines have been developed primarily as live attenuated vaccines derived from genotype II viruses [32,33]. While these vaccines confer effective protection against homologous strains, they provide only limited protection against heterologous or genotypically distinct viruses, including recently reported genotype I/II recombinant strains circulating in Russia, China, and other Asian countries [13,14]. Consequently, these recombinant viruses may continue to spread, even in regions where genotype II viruses are already endemic. In Vietnam, for instance, despite the implementation of a vaccination program approved by the national animal health authority, the prevalence of recombinant viruses appears to be increasing [34]. Under such conditions, the current vaccination strategy may be insufficient and may potentially contribute to further viral recombination. To strengthen preparedness and enable effective responses to emerging threats, rapid detection of recombinant virus incursions is essential. This requires a reliable diagnostic approach capable of genotypic discrimination among circulating ASFV strains. Although several qPCR assays have already been developed for differentiating genotype I, II, and genotype I/II recombinant ASFVs, additional assays targeting distinct conserved genomic regions are needed to improve diagnostic flexibility in response to the ongoing genetic diversification of ASFV. To address this need, a novel duplex qPCR assay targeting *CP2475L* was developed, enabling the precise differentiation of ASFV genotypes I, II, and I/II recombinants within a single reaction.

The assay revealed analytical performance comparable to or exceeding that of previously reported genotype-discriminatory qPCR assays. As summarized in Supplementary Table S7, the reported limits of detection (LODs) of earlier qPCR assays range from approximately 1.18 to 62,600 copies per reaction [16,17,35,36,37,38]. In contrast, the duplex qPCR assay developed in this study achieved an LOD of 10 copies per reaction, with near-perfect amplification efficiency and strong linearity (R^2^ = 0.999 for genotype I- and 0.993 for genotype II), while maintaining precise genotype differentiation (Figure 3 and Figure 4). Moreover, CVs of the duplex qPCR assay ranged from 0.20% to 1.21%, indicating high reproducibility (Table 3). This diagnostic performance supports the assay as a robust and sensitive platform for genotype-specific ASFV detection.

Validation using clinical field samples collected in northern Vietnam in 2025 confirmed the practical applicability of the assay for detecting genotype I/II recombinant ASFV strains. Whole-genome sequencing showed that recombinant viruses circulating in this region exhibited genome-wide accumulation of single-nucleotide substitutions compared with reference genotype I/II recombinant genomes previously reported in China (2021) and Vietnam (2023) [13,15]. However, the *CP2475L* target region remained conserved across all analyzed samples. This conservation, despite ongoing genomic diversification, supports the robustness of the primer and probe design and reinforces the reliability of the assay for detecting currently circulating recombinant variants.

In this duplex qPCR assay, genotype I- and genotype II-specific targets exhibited comparable amplification efficiencies (Figure 3, Table S4). Among clinical samples showing simultaneous amplification of both genotype-specific targets, VN/TH/62/2025 and VN/TH/03/2025 showed ΔCt [Ct _genotype II_ − Ct _genotype I_] values of −5.2 and −6.9, respectively, which were clearly outside this reference range (Table 4). The genotype II-specific target yielded lower Ct values than the genotype I-specific target in these cases; therefore, these samples may represent co-infection with recombinant ASFV and genotype II ASFV. This finding indicates that the assay is also useful for identifying co-infected samples. However, in endemic settings, samples with ΔCt values within the defined reference range may include cases in which genotype I- and genotype II ASFVs are present at comparable levels, as well as cases in which a recombinant virus is co-infected with low levels of genotype I or genotype II virus. Therefore, co-infection cannot be excluded based solely on a ΔCt value within this reference range.

The detection of recombinant viruses with distinct gene deletions indicates that ASFV infections in northern Vietnam are becoming increasingly diverse and complex (Table S6). The co-circulation of multiple viral strains, including vaccine strains, complicates molecular surveillance by reducing the accuracy of detection and interpretation of the epidemiological landscape. In this context, the duplex qPCR assay developed in this study represents a valuable tool for the timely identification of emerging variants and for improving surveillance strategies.

In conclusion, a highly sensitive and specific duplex qPCR assay capable of rapidly differentiating among genotypes I, II, and I/II recombinants was developed. The assay revealed robust performance with field samples and was applicable to the detection of currently circulating recombinant viruses. This diagnostic tool will enhance molecular surveillance and support ASF control efforts in regions experiencing increasing viral genetic diversity.

## Figures and Tables

**Table 1:** Oligonucleotide sequences of primers and probes used in the duplex qPCR assay.

Name	Sequence (5′ → 3′)	Fluorescence Signal	Product Size (bp)
CP2475L-GI-F	CTACCAAATATCTTGCGGCTAATTC	-	199
CP2475L-GI-R	AGAAATCCAGGATTGGTTGTTAAGG	-	
CP2475L-GI-P	CCTCCACCAGCAGGAATACC	FAM	
CP2475L-GII-F	GCGGTTAATTGTGCACCGG	-	318
CP2475L-GII-R	TGTGCGTACTATACGATTGGCTG	-	
CP2475L-GII-P	CCCAGTTTTGAATGGCAGCG	Cy5	

**Table 2:** Information on the reference strains used for primer and probe design in this study.

Number	Strains	Accession Number	Genotype
1	VNUA/rASFV/BG2/2023	PP810979	Recombinant (I + II)
2	VNUA/rASFV/PT2/2023	PP810980	Recombinant (I + II)
3	VNUA/rASFV/VP1/2023	PP810981	Recombinant (I + II)
4	Pig/Henan/123014/2022	OQ504954	Recombinant (I + II)
5	Inner Mongolia/DQDM/2022	OQ504955	Recombinant (I + II)
6	Jiangsu/LG/2021	OQ504956	Recombinant (I + II)
7	NH/P68	NC_044943	I
8	L60	KM262844	I
9	Benin 97/1	AM712239	I
10	E75	NC_044958	I
11	19155_WB	OP312970	I
12	SD/DY-I/2021	MZ945537	I
13	OURT 88/3	NC_044957	I
14	47/Ss/2008	KX354450	I
15	Pig/HeN/ZZ-P1/2021	MZ945536	I
16	141/Nu/1990	MN270974	I
17	AQS-C-1-22	LC659087	II
18	Georgia2007	FR682468	II
19	ASFV-SY18	MH766894	II
20	Pig/HLJ/2018	MK333180	II
21	Kyiv/2016/131	MN194591	II
22	Pol16/20186/o7	MG939583	II
23	Belgium/Etalle/wb/2018	MK543947	II
24	Estonia 2014	LS478113	II

**Table 3:** Reproducibility evaluation of duplex qPCR for intra-assay and inter-assay variation using mixed plasmid standards.

Target Genotype	Template Concentration (Copies/Reaction)	Intra-Assay for Reproducibility				Inter-Assay for Reproducibility			
		Assay Run	Mean	SD	CV (%)	Assay Run	Mean	SD	CV (%)
Genotype I	10^6^	1	20.90	0.09	0.43	3	20.76	0.24	1.15
	10^4^	1	27.79	0.10	0.37	3	27.62	0.20	0.71
	10^2^	1	34.72	0.33	0.96	3	34.51	0.42	1.21
Genotype II	10^6^	1	21.34	0.06	0.29	3	21.23	0.24	1.15
	10^4^	1	28.30	0.06	0.20	3	28.26	0.08	0.28
	10^2^	1	35.30	0.35	0.99	3	35.18	0.37	1.06

**Table 4:** ASFV genotypes detected in samples collected from northern Vietnam between July and October 2025.

Sample Name	Sampling Site	Sample Collection Date	ASFV Screening qPCR *	Duplex qPCR *		Decision
				Genotype I	Genotype II	
VN/TQ/97/2025	Tuyen Quang	5 September 2025	21.3	26.5	27.8	Recombinant (I + II)
VN/LS/100/2025	Lang Son	8 September 2025	16.7	16.5	17.1	Recombinant (I + II)
VN/BG/122/2025	Bac Giang	22 October 2025	26.5	38.1	26.3	Genotype II
VN/NA/113/2025	Nghe An	23 September 2025	–	NT	NT	negative
VN/NA/114/2025	Nghe An	23 September 2025	–	NT	NT	negative
VN/HT/115/2025	Ha Tinh	10 October 2025	23.6	38.3	24.1	Genotype II
VN/ND-NB/116/2025	Ninh Binh	16 October 2025	–	NT	NT	negative
VN/NA/117/2025	Nghe An	16 October 2025	20.3	18.5	19.3	Recombinant (I + II)
VN/NA/118/2025	Nghe An	16 October 2025	18.5	18.4	19.2	Recombinant (I + II)
VN/TH/62/2025	Thanh Hoa	25 July 2025	20.0	24.2	19.0	Co-infection
VN/TH/03/2025	Thanh Hoa	25 July 2025	20.4	26.5	19.6	Co-infection
VN/LS/06/2025	Lang Son	29 July 2025	18.0	18.0	18.5	Recombinant (I + II)
VN/LS/08/2025	Lang Son	29 July 2025	22.9	22.2	22.9	Recombinant (I + II)
VN/PT/20/2025	Phu Tho	30 July 2025	21.1	21.0	21.6	Recombinant (I + II)
VN/PT/24/2025	Phu Tho	30 July 2025	21.4	21.0	21.6	Recombinant (I + II)
VN/PT/31/2025	Phu Tho	30 July 2025	18.0	18.2	18.6	Recombinant (I + II)
VN/PT/33/2025	Phu Tho	30 July 2025	25.8	25.0	25.6	Recombinant (I + II)
VN/BN/124/2025	Bac Ninh	27 October 2025	16.2	16.0	16.5	Recombinant (I + II)
VN/PT/21/2025	Phu Tho	30 July 2025	22.0	23.0	23.8	Recombinant (I + II)
VN/LS/102/2025	Lang Son	8 September 2025	21.9	21.2	21.9	Recombinant (I + II)

NT, not tested; –, not detected. * Ct values are indicated. Ct values above the detection threshold are shown in gray font and were not considered positive.

## Data Availability

The genome sequence data of genotype I/II recombinant ASFVs have been deposited in GenBank under accession numbers LC921664–LC921673. The datasets generated and/or analyzed during the current study are available from the corresponding author upon reasonable request.

## Supplementary Materials

The following supporting information can be downloaded at https://www.mdpi.com/article/10.3390/v18060677/s1: Table S1: Information on the reference strains used to evaluate the conservation of the primer and probe sequences in this study. Table S2: Primer sets for tiled PCR amplification of the ASFV whole genome. Table S3: Ct values obtained from tenfold serial dilutions of genotype I- and II standard plasmids. Table S4: ΔCt values between genotype I- and genotype II-specific targets using a synthetic recombinant control. Table S5: Whole-genome sequence identity between 2023 and 2025 recombinant strains from Vietnam. Table S6: Mutations identified in the genomes of 10 ASFV samples classified as genotype I/II recombinant by the duplex qPCR assay. Table S7: Limits of detection (LOD) of genotype-discriminatory qPCR assays for ASFV [16,17,35,36,37,38].

## Abbreviations

The following abbreviations are used in this manuscript:

ASF	African swine fever
ASFV	African swine fever virus
qPCR	quantitative polymerase chain reaction
LOD	Limit of detection
